# Anti-osteosarcoma effect of antiserum against cross antigen TPD52 between osteosarcoma and *Trichinella spiralis*

**DOI:** 10.1186/s13071-021-05008-6

**Published:** 2021-09-26

**Authors:** Tao-Tao Yue, Nan Zhang, Jian-Hua Li, Xiang-Yun Lu, Xiao-Cen Wang, Xin Li, Hong-Bo Zhang, Shu-Qin Cheng, Bo-Bo Wang, Peng-Tao Gong, Xi-Chen Zhang

**Affiliations:** Key Laboratory of Zoonosis Research By Ministry of Education, Institute of Zoonosis, College of Veterinary Medicine, Jilin University, Changchun, 130062 China

**Keywords:** *Trichinella spiralis*, Antitumour effect, Cross antigens, Tumour protein D52, Apoptosis

## Abstract

**Background:**

*Trichinella spiralis* (*T. spiralis*) is a parasite occurring worldwide that has been proven to have antitumour ability. However, studies on the antitumour effects of cross antigens between the tumour and *T. spiralis* or antibodies against cross antigens between tumours and *T. spiralis* are rare.

**Methods:**

To study the role of cross antigens between osteosarcoma and *T. spiralis,* we first screened the cDNA expression library of *T. spiralis* muscle larvae to obtain the cross antigen gene *tumour protein D52* (*TPD52*), and prepared fusion protein TPD52 and its antiserum. The anti-osteosarcoma effect of the anti-TPD52 antiserum was studied using cell proliferation and cytotoxicity assays as well as in vivo animal models; preliminary data on the mechanism were obtained using western blot and immunohistochemistry analyses.

**Results:**

Our results indicated that TPD52 was mainly localized in the cytoplasm of MG-63 cells. Anti-TPD52 antiserum inhibited the proliferation of MG-63 cells and the growth of osteosarcoma in a dose-dependent manner. The tumour inhibition rate in the 100 μg treatment group was 61.95%. Enzyme-linked immunosorbent assay showed that injection of anti-TPD52 antiserum increased the serum levels of IFN-γ, TNF-α, and IL-12 in nude mice. Haematoxylin and eosin staining showed that anti-TPD52 antiserum did not cause significant pathological damage. Apoptosis of osteosarcoma cells was induced by anti-TPD52 antiserum in vivo and in vitro.

**Conclusions:**

Anti-TPD52 antiserum exerts an anti-osteosarcoma effect by inducing apoptosis without causing histopathological damage.

**Graphical abstract:**

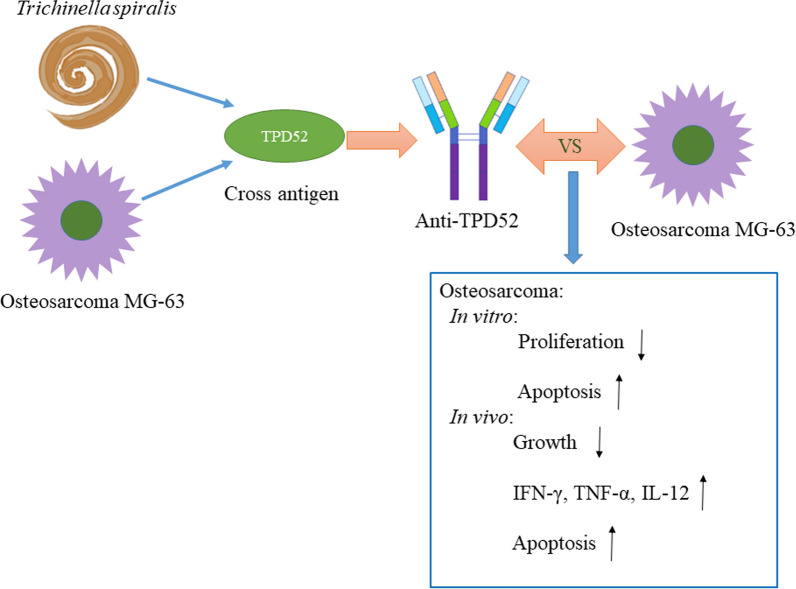

**Supplementary Information:**

The online version contains supplementary material available at 10.1186/s13071-021-05008-6.

## Background

Malignant tumours are a significant threat to human health and a leading cause of death worldwide [1]. To date, ideal prevention and treatment methods for malignant tumours, including osteosarcoma, have not been found. Surgery, radiotherapy, and chemotherapy are conventional therapies, but they have severe side effects [2], underscoring the need for novel therapeutic approaches.

In recent years, the relationship between parasites and tumours has become a research hotspot, with several studies confirming a negative correlation between certain parasitic infections and tumours [3, 4]. Studies have confirmed that *Echinococcus granulosus* [5, 6], *Trichomonas vaginalis* [7, 8], *Trypanosoma cruzi* [9, 10], *Toxoplasma gondii* [11, 12], and *Plasmodium* spp. [13–15] can inhibit the growth of a variety of tumours or prolong the survival of tumour-bearing animals. Research has shown that cross antigens between tumours and these parasites, such as certain carbohydrate antigens like Tn and Tk, act via critical molecular mechanisms generating antitumour effects and are thought to be one of the important mechanisms by which parasites inhibit tumour growth [16, 17]. These antigens, also called heterogenetic antigens, can break immune tolerance and demonstrate good immunogenicity [18]. Therefore, these parasites and their antigens offer a new direction for cancer biotherapy.

*Trichinella spiralis*, a food-borne parasite with a worldwide distribution, has powerful immunomodulatory properties and can induce antitumour immunity [19–21]. However, the exact mechanism by which *T. spiralis* exerts antitumour effects remains unclear. Previous studies have indicated that *T. spiralis* infection significantly improved survival rates in mice with breast cancer [22] and inhibited the progression of B16F10 melanoma [23, 24] and murine forestomach carcinoma [25]. *Trichinella spiralis* muscle larvae (ML) excretory-secretory antigens (ESAs) suppressed the proliferation of H446 [26] and A549 cells [27] in a time- and dose-dependent manner. Despite good antitumour effects, however, because *T. spiralis* is a pathogen, the direct use of *T. spiralis* as a therapeutic agent not only causes aversion and psychological rejection but also brings the risk of disease. Therefore, the development of effective *T. spiralis* antitumour active substances is critically needed.

Several cross antigens between tumours and *T. spiralis* have been reported recently. Immunizing mice with a cross antigen between myeloma and *T. spiralis* resulted in a 51% tumour inhibition rate [28]. However, most animal models of human tumours employ immunodeficient animals, and immunization with antigens cannot induce effective antitumour immunity. Treatment with antibodies is an alternative strategy, but the antitumour effects of antibodies against cross antigens between tumours and *T. spiralis* have rarely been reported.

In this study, cross antigens between human osteosarcoma and *T. spiralis* were obtained by screening a cDNA expression library. TPD52 antigen was selected, its antiserum was prepared, and its anti-osteosarcoma effect was examined.

## Methods

### Mice, cell lines, and parasites

Female BALB/c nude mice (4–6 weeks old) were purchased from Beijing HuaFuKang Biotechnology, China (Production License No. SCXK [Jing] 2019-0008). Human osteosarcoma MG-63 cells (purchased from the Cell Bank of the Chinese Academy of Sciences) were cultured in minimum essential medium (Boster, Shanghai, China), containing 10% (v/v) fetal bovine serum (BI, Shanghai, China) and 100 μg of penicillin–streptomycin, at 37 °C in a humidified incubator with 5% CO_2_. *Trichinella spiralis* (ISS 534) were maintained by serial passage in Wistar rats.

### Screening of cross antigens between osteosarcoma and *T. spiralis*

The cDNA expression library of *T. spiralis* ML was constructed and preserved in our laboratory [29]. The *T. spiralis* and MG-63 cell antigens were obtained by sonication, and their concentrations were measured using a BCA protein assay kit (BCA, Thermo Fisher Scientific, MA, USA). Mice were subcutaneously inoculated with these two antigens emulsified in complete Freund's adjuvant (CFA, Sigma-Aldrich, St. Louis, MO, USA) and boosted after 2 and 3 weeks. The serum antibody titre was measured by enzyme-linked immunosorbent assay (ELISA).

XL-1 Blue *Escherichia coli* (*E. coli*) antigens were obtained by sonication, added to the anti-MG-63 cell antiserum at a ratio of 1:2, and incubated overnight at 4 °C to remove antibodies having cross-reactivity with *E. coli* antigens.

XL1-Blue *E. coli* was resuspended in sterile 10 mM MgSO_4_, incubated with SM buffer and the phage library, then mixed with NZY top agar and incubated on NZY agar for 6 h. The expression of recombinant protein was induced by isopropyl β-d-thiogalactoside (IPTG)-treated nitrocellulose membrane at 37 °C for 6 h. After blocking with 5% non-fat dry milk, the membrane was incubated with anti-MG-63 cell antiserum (diluted at 1:500) and horseradish peroxidase (HRP)-conjugated goat anti-mouse immunoglobulin G (IgG; diluted at 1:5000) in sequence. The antigen–antibody complex was visualized by staining with diaminobenzidine tetrahydrochloride. The positive plaques were dissolved and diluted with SM buffer and subjected to immune rescreening until consistent results were obtained. Using the positive plaques dissolved in SM buffer as a template, primers P1 and P2 were used to amplify the inserted gene fragments. The amplified products were then sequenced, and the DNA and amino acid sequences of the screened gene were obtained by comparison with the BLAST database. DNASTAR software was used to determine the open reading frame (ORF) of genes and the isoelectric point and molecular weight of proteins. ProtParam tools were used to analyse the hydrophilicity of proteins and VaxiJen 2.0 was used to predict antigenicity.

### Preparation of antiserum

Using the *T. spiralis* cDNA generated in our laboratory as a template, the *TPD52* gene was amplified by PCR and inserted into pET-32a vector to construct a recombinant plasmid pET-32a-*TPD52*. Primers are shown in Table 1. The recombinant plasmid was transformed into *E. coli* BL21 (Tiangen Biotech, Beijing, China). After inducing expression with IPTG and purification by Ni purification column, the fusion protein TPD52 was identified using HRP-conjugated 6× His tag antibody (HRP-66005, Proteintech, Wuhan, China) and mouse anti-*T. spiralis* antiserum. The fusion protein TPD52 concentration was determined using a BCA assay. Mice were inoculated subcutaneously with 100 μg of fusion protein TPD52 emulsified in CFA and boosted after 2 and 3 weeks. The serum antibody titre was measured by ELISA and its concentration was determined using a BCA assay.

**Table 1:** Primer sequences employed in this study

Primer	Primer sequences (5′ → 3′ direction)	Usage
P1	ATACGACTCACTATAGGGCGAATTGGC	cDNA amplification
P2	CTCGGGAAGCGCGCCATTGTGTTGGT	
P3	*AGTACT*ATGGAGAATCGAACTACAGAA	*TPD52* amplification
P4	*TCTAGA*TCATTCAAATTTGTTTTCTAC	

Italicized sequences represent the restriction sites ScaI and XbaI

### Immunofluorescence assay

MG-63 cells were seeded into 24-well plates and cultured overnight and then fixed using methanol, permeabilized with 0.5% Triton X-100, and blocked with 5% skimmed milk powder. The cells were incubated overnight in anti-TPD52 antiserum as the primary antibody, and then treated with fluorescein isothiocyanate (FITC)-conjugated goat anti-mouse IgG (Proteintech, Wuhan, China). The cells were then stained with DAPI (Boster, Shanghai, China) and observed using a confocal laser microscope (FV1000, Olympus, Japan).

### Cell proliferation and cytotoxicity assays

MG-63 cells were incubated with anti-TPD52 antiserum (5 μg, 10 μg, 25 μg, 50 μg, and 100 μg) for 48 h. Since etoposide is a commonly used anti-osteosarcoma drug in clinical practice [30], it was selected as the positive control and used at a concentration of 50 and 100 μM. Cell proliferation was determined by Cell Counting Kit-8 (CCK-8, Trans, Beijing, China) assay at 450 nm wavelength. The cell proliferation rate was calculated as (OD treatment − OD blank)/(OD control − OD blank) × 100%.

Anti-TPD52 antiserum-treated MG-63 cell culture supernatant was collected, and the cytotoxicity was determined by lactate dehydrogenase (LDH) assay (Beyotime, Shanghai, China) according to the manufacturer's protocol. The percentage of LDH release was calculated as (LDH treatment − LDH control)/LDH total lysis − LDH control) × 100%.

### Animal experimental models

Female BALB/c nude mice were randomly subdivided into seven groups of five animals each. One group was not challenged with osteosarcoma and was set up as an untreated control (NC) group, and the other six groups were injected subcutaneously with 2 × 10^6^ MG-63 cells into the armpits of the forelimbs. When the tumour was palpable, anti-*T. spiralis* antiserum (100 μg) or anti-TPD52 antiserum (25, 50, and 100 µg) was continuously injected subcutaneously around the tumour daily for 21 days. The same volume of phosphate-buffered saline (PBS) and negative serum was applied in the osteosarcoma model group and negative control (NS) group, respectively. Tumour volume was calculated according to the following formula: volume (mm^3^) = *R*/2 × *r*^2^, where *R* and *r* are the longest and the shortest diameters, respectively. The inhibition rate was evaluated using the equation.

Inhibition rate = (1 − *T*/*C*) × 100%, where *T* and *C* are the average tumour volume and weight in the experimental and PBS groups, respectively.

### Cytokine assays

Blood was collected from each group of nude mice, serum was separated, and interleukin-2 (IL-2), IL-4, IL-6, IL-10, IL-12, interferon gamma (IFN-γ), and tumour necrosis factor alpha (TNF-α) were determined using an ELISA kit (Invitrogen, CA, USA). All operations were performed according to the manufacturer's instructions.

### Histopathological changes

The heart, liver, spleen, lung, and kidneys of each group of nude mice were harvested for haematoxylin and eosin (HE) staining, and the histopathological damage was observed under a microscope. The heart, liver, spleen, lung, and kidneys were scored using the histopathological scoring scale used by Wu [31], Camargo [32], Schwab [33], Kredel [34], and Jha [35]. The score was positively correlated with pathological changes. Scoring details are presented in the supplementary material (Additional file 1: Table S1).

### Anti-osteosarcoma mechanisms

The role of apoptosis in reducing tumour growth was investigated by examining apoptosis-associated proteins in western blot analyses. MG-63 cells were incubated with anti-TPD52 antiserum (25, 50, and 100 μg) for 24 h. Cells were lysed using radioimmunoprecipitation assay (RIPA) buffer, and total lysates were collected, separated by electrophoresis, and transferred to polyvinylidene fluoride (PVDF) membranes (Millipore, MA, USA) to quantify protein content by incubating with primary antibody and HRP-conjugated goat anti-mouse IgG. The immunoreactive bands were detected by enhanced chemiluminescence (ECL; Millipore, MA, USA), according to the manufacturer’s instructions. The primary antibodies used were anti-BAX polyclonal antibody (50599-2-Ig, Proteintech), anti-BCL-2 polyclonal antibody (26593-1-AP, Proteintech), anti-cleaved caspase-3 polyclonal antibody (ab2302, Abcam, MA, USA), and anti-β-actin monoclonal antibody (66009-1-Ig, Proteintech).

Immunohistochemical staining was performed on tumour tissues in each group to observe the expression of BCL-2. The positive intensities were determined using ImageJ software.

### Statistics

Comparisons between the control and experimental groups were performed using Prism 7.0 software (GraphPad Software, Inc.) and are described as mean ± standard deviation (M ± SD). Analysis of variance (ANOVA) was used and significance was indicated by *P < 0.05, **P < 0.01 ***P < 0.001, or ****P < 0.0001.

## Results

### The presence of cross antigens between osteosarcoma and *T. spiralis*

**Fig. 1:**
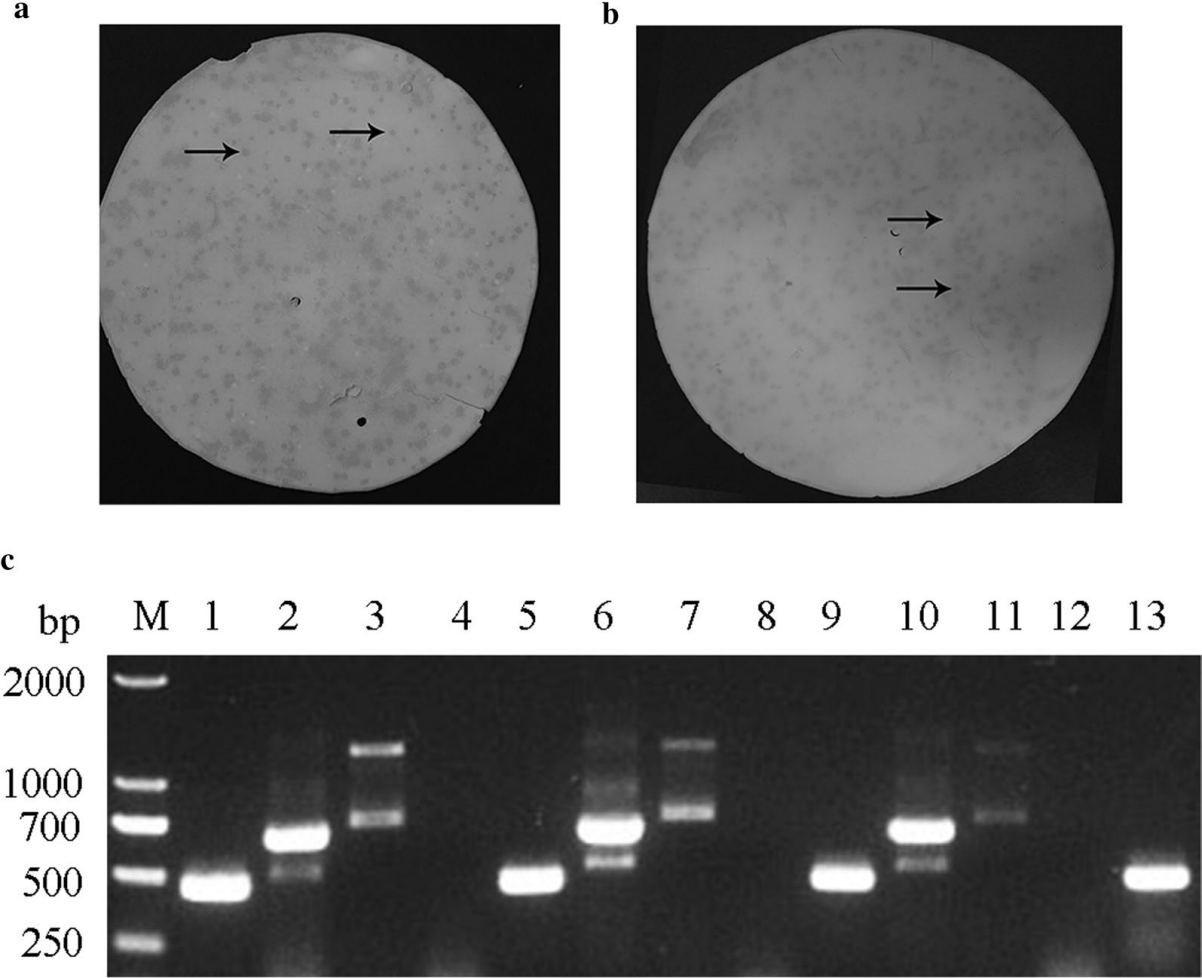
Screening and identification of cross antigen genes between osteosarcoma and *T. spiralis*. The cDNA expression library of *T. spiralis* muscle larva was primary (**a**) and secondary (**b**) screened by anti-MG-63 cell antiserum. Arrows represent positive plaque. PCR using thirteen positive plaque solution as a template to obtain cross antigen genes between osteosarcoma and *T. spiralis* (**c**)

Thirteen positive plaques were obtained by screening the cDNA expression library of *T. spiralis* ML twice with anti-MG-63 cell antiserum (Fig. 1a, b). The positive plaque solution was used as a template for PCR to obtain multiple gene fragments of different sizes, ranging from 250 to 2000 base pairs (bp) (Fig. 1c). Seven cross antigen genes were identified by sequencing and BLAST analysis (Additional file 2: Table S2). One of these genes (XM_003375331.1) encodes TPD52 with a 459-bp ORF encoding 152 amino acids, with a predicted molecular weight of 17 kDa. When the grand average of hydropathicity of the protein predicted by ProtParam tools is negative, the protein is hydrophilic. Among the seven cross antigens, the grand average of hydropathicity of TPD52 was -0.767 (Additional file 2: Table S2), indicating that the hydrophilicity of TPD52 was the highest. When the prediction result of VaxiJen 2.0 is greater than the threshold of 0.5, it indicates that the protein is a potential antigen. When targeting tumours, the predicted score of TPD52 is 0.7106 (Additional file 2: Table S2), indicating that this protein may be the most protective. Hence, this gene was selected for subsequent experiments.

### Expression and purification of cross antigen TPD52 and preparation of antiserum

**Fig. 2:**
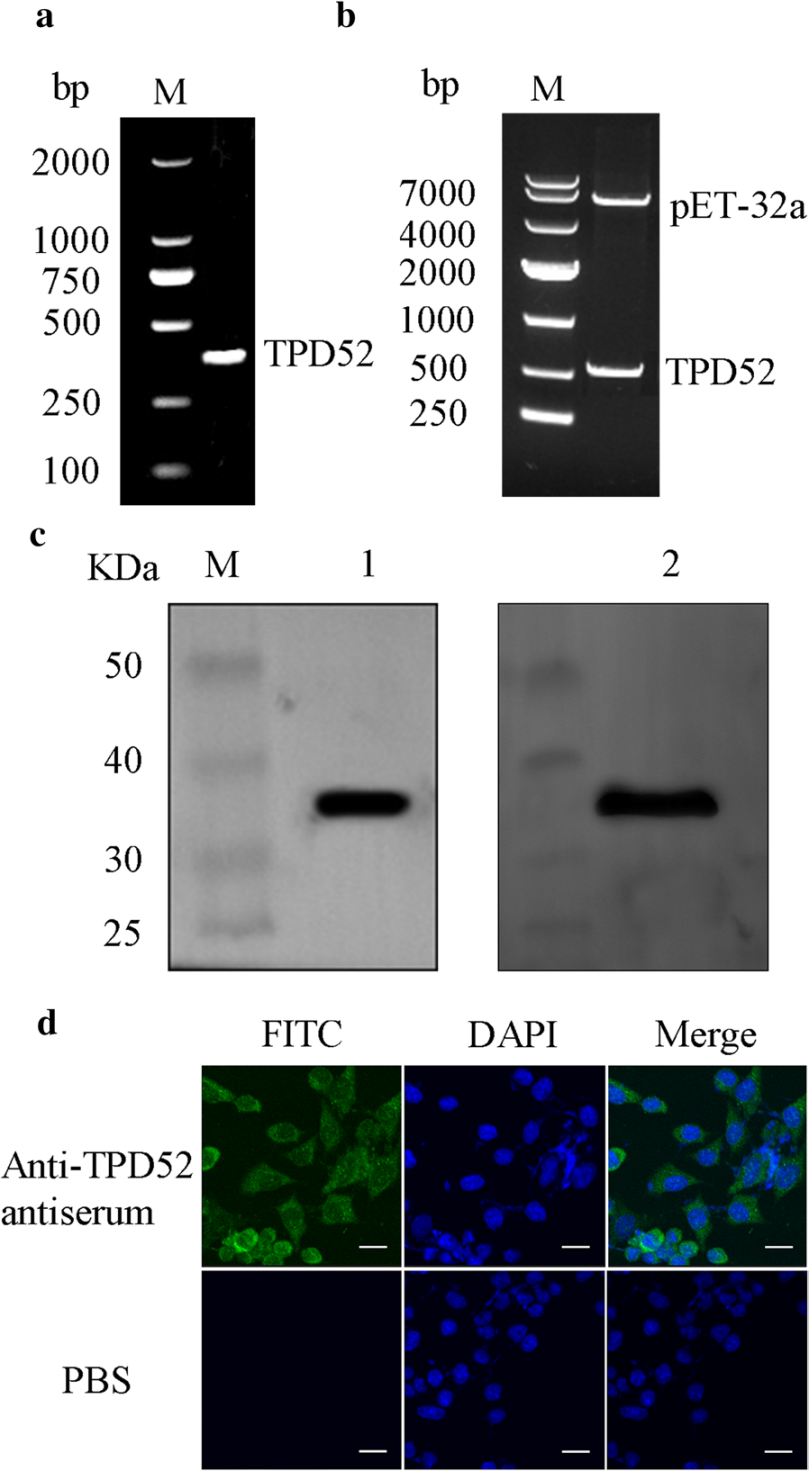
The localization of TPD52 in MG-63 cells. Using *T. spiralis* cDNA as a template, the *TPD52* gene was amplified by PCR and identified (**a**). The recombinant pET-32a-*TPD52* plasmid was characterized by restriction digestion (ScaI and XbaI) (**b**). The purified fusion protein TPD52 was identified by western blotting using HRP-conjugated 6× His tag antibody (Lane 1) and mouse anti-*T. spiralis* antiserum as primary antibodies (Lane 2) (**c**). TPD52 in MG-63 cells were detected by laser confocal microscopy using mouse anti-TPD52 antiserum as the primary antibody. Scale bars: 20 µm (**d**)

A 459-bp gene was amplified by PCR, which was consistent with expectations (Fig. 2a). It was inserted into the expression plasmid pET-32a and transformed into competent *E. coli* BL-21 cells. Restriction digestion and sequencing showed that the expression vector was successfully constructed (Fig. 2b). Western blot revealed that the size of the fusion protein TPD52 was approximately 36 kDa and that it could bind specifically to the mouse anti-*T. spiralis* antiserum (Fig. 2c).

Mice were inoculated subcutaneously with fusion protein TPD52 to generate anti-TPD52 antiserum. Serum was collected 7 days after the last immunization. ELISA results showed that the titre of the anti-TPD52 antiserum was 1:102400 (Additional file 3: Table S3).

### TPD52 is mainly localized in the cytoplasm

After MG-63 cells were fixed, permeabilized, and blocked, anti-TPD52 antiserum was used as the primary antibody to detect the localization of TPD52 in MG-63 cells. Laser confocal microscopy results revealed that green fluorescence existed mainly in the cytoplasm (Fig. 2d), providing evidence that cross antigen TPD52 between osteosarcoma and *T. spiralis* was localized in the cytoplasm of MG-63 cells.

### Anti-TPD52 antiserum reduced the proliferation of MG-63 cells

**Fig. 3:**
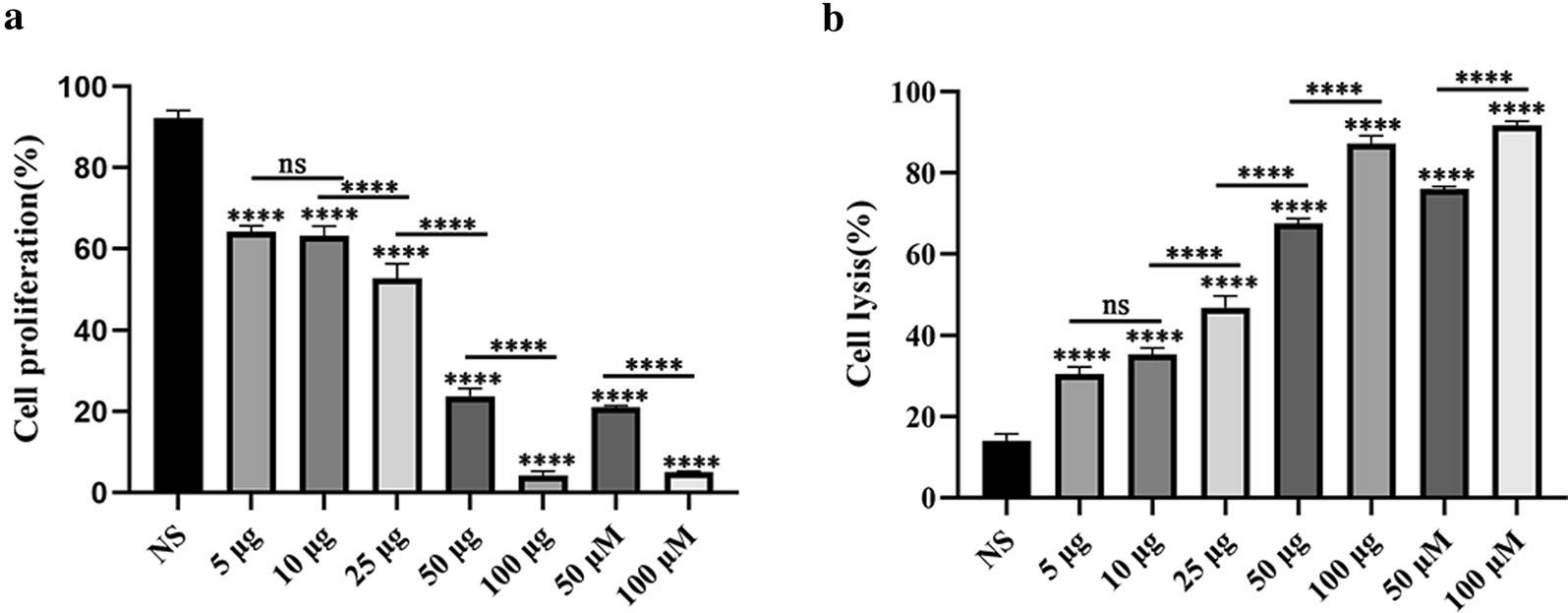
Effects on the proliferation and toxicity of MG-63 cells treated with anti-TPD52 antiserum for 48 h. CCK-8 assay was used to test the effect of anti-TPD52 antiserum on MG-63 cell proliferation (**a**). LDH assay was used to detect the cytotoxicity of anti-TPD52 antiserum to MG-63 cells (**b**). NS: negative serum. 5 μg, 10 μg, 25 μg, 50 μg, and 100 μg: anti-TPD52 antiserum. 50 μM and 100 μM: etoposide as positive control (****P < 0.0001; ns: non-significant)

CCK-8 and LDH assays were conducted to evaluate the proliferation of MG-63 cells. The results showed that etoposide inhibited the proliferation of MG-63 cells in a dose-dependent manner (ANOVA, *F*_(7, 16)_ = 860.4, *P* < 0.0001; Fig. 3a) and was cytotoxic to MG-63 cells in a dose-dependent manner (ANOVA, *F*_(7, 16)_ = 827, *P* < 0.0001; Fig. 3b). The cell proliferation decreased with increasing anti-TPD52 antiserum concentration, indicating that the inhibitory effect of anti-TPD52 antiserum on MG-63 cells was dose-dependent (ANOVA, *F*_(7, 16)_ = 860.4, *P* < 0.0001; Fig. 3a). The results of the LDH assay also showed dose-dependent cytotoxicity of the anti-TPD52 antiserum on MG-63 cells (ANOVA, *F*_(7, 16)_ = 827, *P* < 0.0001; Fig. 3b). These results revealed that the anti-TPD52 antiserum inhibits the proliferation of MG-63 cells in a dose-dependent manner.

### Anti-TPD52 antiserum inhibited the growth of osteosarcoma

**Fig. 4:**
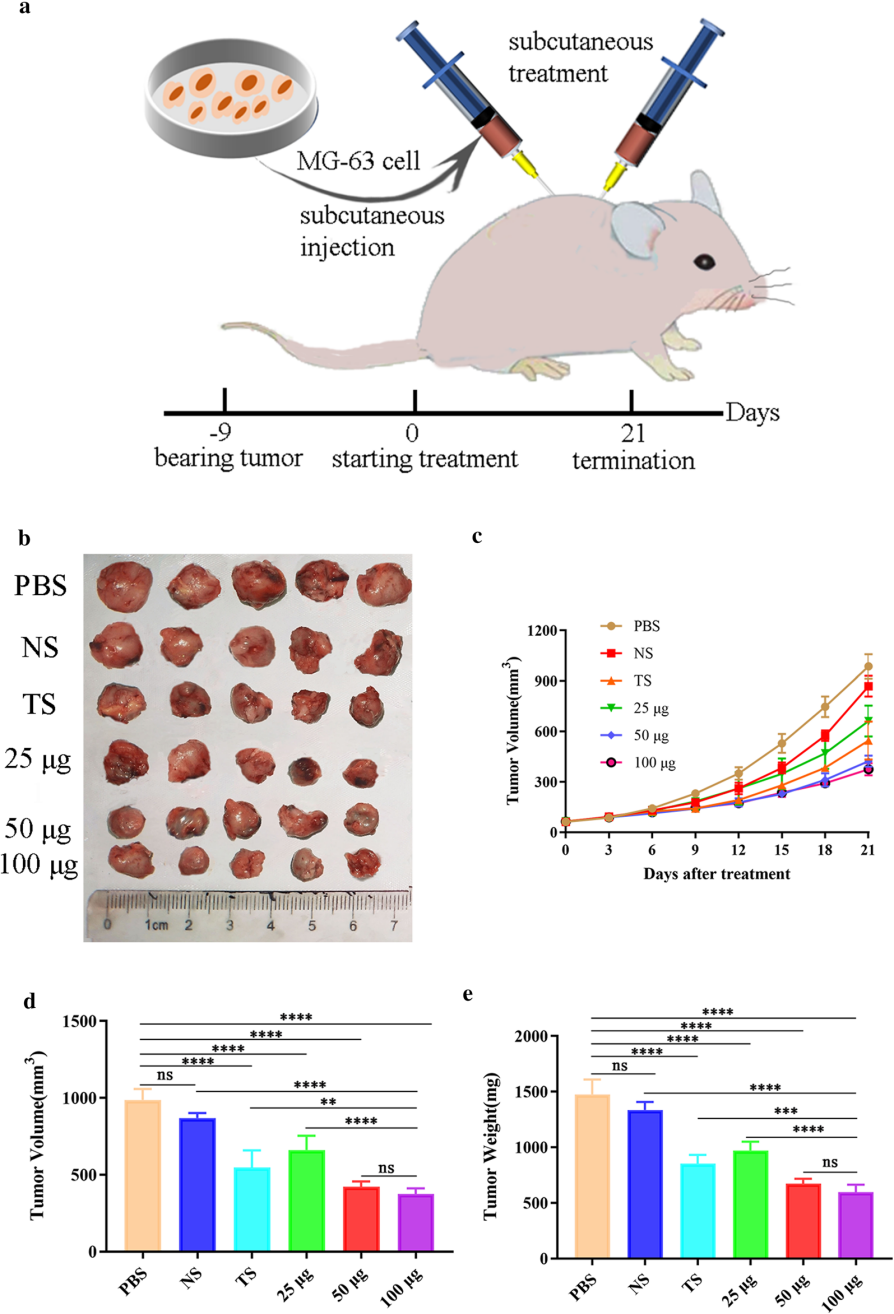
Anti-osteosarcoma effect of anti-TPD52 antiserum in vivo. Schematic diagram of the experimental process. In brief, after the osteosarcoma challenge, treatment was started on day 9 with subcutaneous injections around the tumour; the trial was terminated on the 21st day (**a**). Osteosarcoma of nude mice from each group (**b**). The growth trend of osteosarcoma (**c**). Osteosarcoma volume in nude mice in each group (**d**). Osteosarcoma weight in nude mice in each group (**e**). *Abbreviations*: PBS: osteosarcoma model. NS: negative serum. TS: 100 µg anti-*T. spiralis* antiserum. 25 μg, 50 μg, and 100 μg: anti-TPD52 antiserum (**P < 0.01; ***P < 0.001; ****P < 0.0001; ns: non-significant)

Osteosarcoma-bearing nude mice were used to test the effect of anti-TPD52 antiserum in vivo. The treatment schedule is shown in Fig. 4a. The effects of anti-TPD52 antiserum on osteosarcoma were assessed by determining the size and weight of tumours. The results showed that anti-*T. spiralis* antiserum inhibited the growth of osteosarcoma, with average tumour volume and weight inhibition rates of 44.58% and 42.14%, respectively. In addition, anti-TPD52 antiserum reduced the volume (ANOVA, *F*_(5, 24)_ = 60.14, *P* < 0.0001; Fig. 4b–d) and weight (ANOVA, *F*_(5, 24)_ = 91.57, *P* < 0.0001; Fig. 4e) of osteosarcoma in a dose-dependent manner, indicating that antiserum inhibits the growth of osteosarcoma. The most significant effects were observed in the 50 μg and 100 μg groups, wherein the average tumour volume inhibition rates were 57.08% and 61.95%, and the average tumour weight inhibition rates were 54.38% and 59.56%, respectively (Additional file 4: Table S4).

### Anti-TPD52 antiserum regulates the secretion of cytokines

**Fig. 5:**
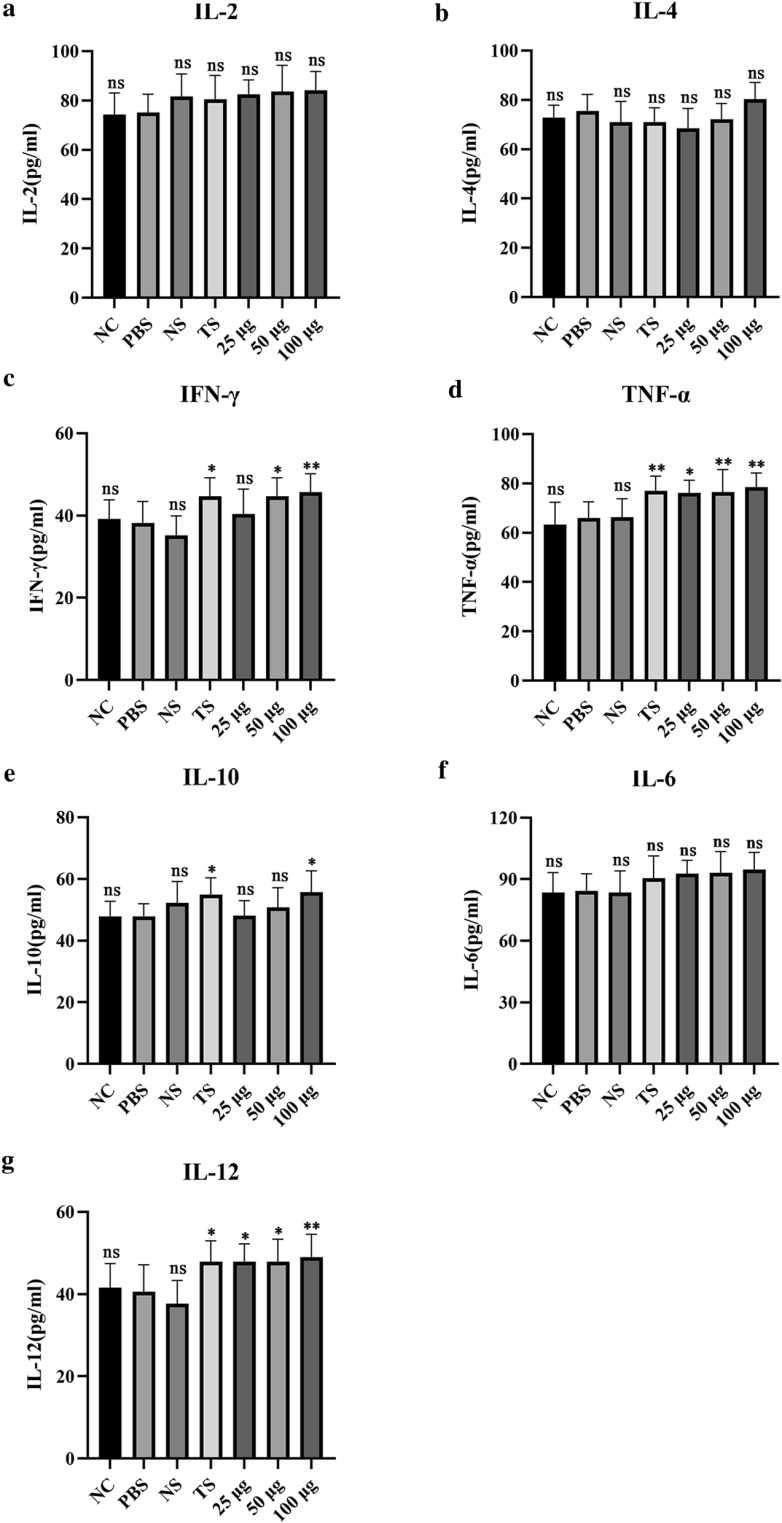
Changes in the serum cytokine levels in nude mice. IL-2 (**a**). IL-4 (**b**). IFN-γ (**c**). TNF-α (**d**). IL-10 (**e**). IL-6 (**f**). IL-12 (**g**). NC: untreated control. PBS: osteosarcoma model. NS: negative serum. TS: 100 µg anti-*T. spiralis* antiserum. 25 μg, 50 μg and 100 μg: anti-TPD52 antiserum (*P < 0.05; **P < 0.01; ns: non-significant)

ELISA results showed that there were no significant differences in IL-2 (ANOVA, *F*_(6, 63)_ = 2.164, *P* = 0.0583; Fig. 5a), IL-4 (ANOVA, *F*_(6, 63)_ = 3.225, *P* = 0.0079; Fig. 5b), and IL-6 (ANOVA, *F*_(6, 63)_ = 2.735, *P* = 0.0200; Fig. 5f) levels in any group. Anti-*T. spiralis* antiserum increased the levels of IFN-γ (ANOVA, *F*_(6, 63)_ = 6.601, *P* < 0.0001; Fig. 5c), TNF-α (ANOVA, *F*_(6, 63)_ = 8.062, *P* < 0.0001; Fig. 5d), IL-10 (ANOVA, *F*_(6, 63)_ = 3.402, *P* = 0.0057; Fig. 5e), and IL-12 (ANOVA, *F*_(6, 63)_ = 6.833, *P* < 0.0001; Fig. 5g) in the serum of nude mice. More importantly, different doses of anti-TPD52 antiserum increased the levels of IL-12 (ANOVA, *F*_(6, 63)_ = 6.833, *P* < 0.0001; Fig. 5g), IFN-γ (ANOVA, *F*_(6, 63)_ = 6.601, *P* < 0.0001; Fig. 5c), and TNF-α (ANOVA, *F*_(6, 63)_ = 8.062, *P* < 0.0001; Fig. 5d). However, the level of IL-10 was increased only in 100 μg groups (ANOVA, *F*_(6, 63)_ = 3.402, *P* = 0.0057; Fig. 5e).

### Anti-TPD52 antiserum did not cause distinct histopathological changes

**Fig. 6:**
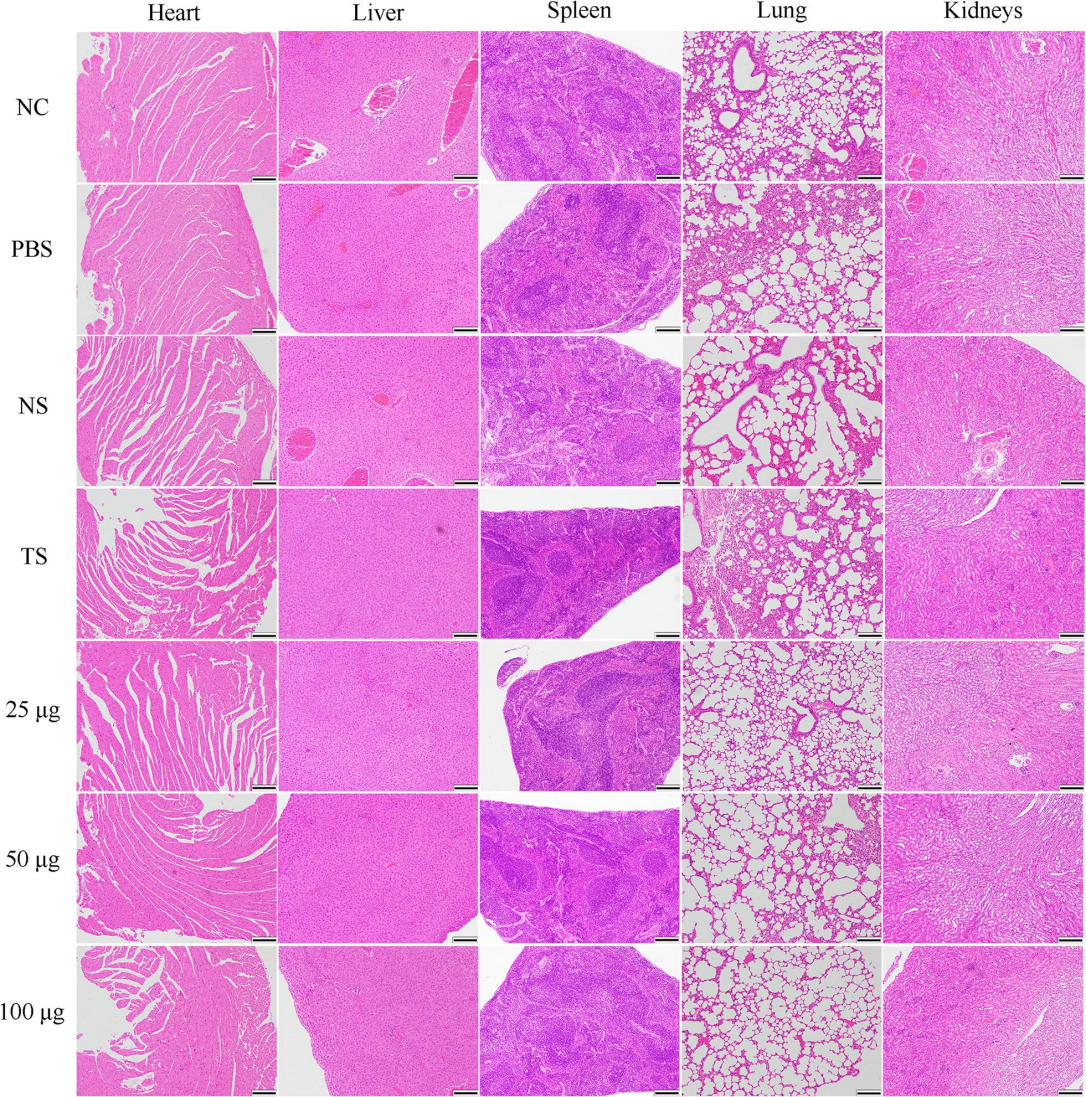
HE staining of nude mice tissues and organs (×100). NC: untreated control. PBS: osteosarcoma model. NS: negative serum. TS: 100 µg anti-*T. spiralis* antiserum. 25 μg, 50 μg, and 100 μg: anti-TPD52 antiserum. Scale bars: 20 µm

HE staining was conducted on the heart, liver, spleen, lung, and kidneys of each group to examine whether the anti-TPD52 antiserum caused any histopathological damage. HE staining showed that the myocardial structure of the mice in each group was normal and arranged tightly, and there was no edema or necrosis. The hepatic lobules were intact, and the morphology of the liver cells was normal, without a large amount of lymphocyte infiltration, vasodilatation, and spotty or focal necrosis. The spleen showed no excessive lymphocyte and macrophage infiltration or fibrous tissue proliferation, and no nodule formation. The alveolar wall was intact. There was no abnormal exudate in the lung interstitium, and no obvious congestion, edema, or red blood cell exudation. The structure of the glomerulus was normal. There was no turbidity and swelling of the renal tubular epithelium, the cell boundaries were clear, and there was no blood stasis, edema, or necrosis in the interstitium (Fig. 6). The results showed that the anti-TPD52 antiserum did not cause significant pathological damage to tissues and organs.

### Anti-osteosarcoma effects of anti-TPD52 antiserum were associated with apoptosis

**Fig. 7:**
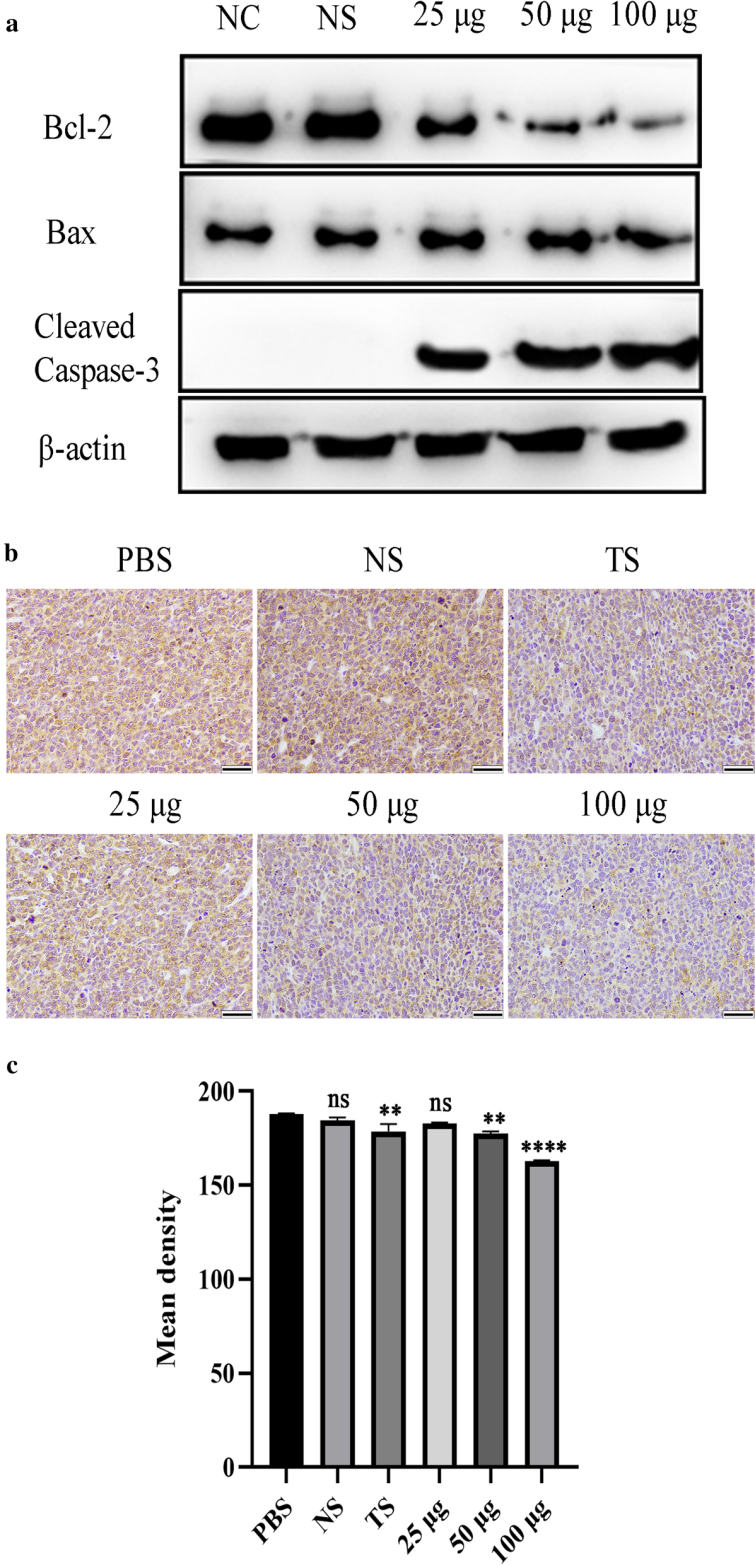
Detection of apoptosis after treatment with anti-TPD52 antiserum. Western blot was used to detect the expression of apoptosis-related proteins (**a**). Detection of BCL-2 expression in osteosarcoma by immunohistochemistry (×400). Scale bars: 5 µm (**b**). BCL-2 expression was quantified using ImageJ software (**c**). NC: untreated control. PBS: osteosarcoma model. NS: negative serum. TS: 100 µg anti-*T. spiralis* antiserum. 25 μg, 50 μg and 100 μg: anti-TPD52 antiserum (**P < 0.001; ****P < 0.0001; ns: non-significant)

Many studies have shown that *T. spiralis* and its proteins can exert antitumour effects by inducing apoptosis [21, 36, 37]. This study monitored the expression of cleaved caspase-3, BCL-2, and BAX in MG-63 cells to explore whether apoptosis is involved in the death of MG-63 cells induced by anti-TPD52 antiserum. The results showed that the expression of BAX did not change significantly. The activation of caspase-3 was detected after treatment with anti-TPD52 antiserum, and the expression of BCL-2 was significantly downregulated in a dose-dependent manner (Fig. 7a).

Immunohistochemistry was used to analyse the expression of BCL-2 in osteosarcoma. The results showed that BCL-2 was localized in the cytoplasm and its expression was decreased in osteosarcoma treated with anti-*T. spiralis* antiserum. Anti-TPD52 antiserum also reduced the expression of BCL-2 in osteosarcoma in a dose-dependent manner (ANOVA, *F*_(5, 6)_ = 44.29, *P* = 0.0001; Fig. 7b, c), indicating that the anti-TPD52 antiserum could induce apoptosis in osteosarcoma.

## Discussion

The current study establishes that the antitumour mechanisms of worms such as *T. spiralis* are based mainly on the following three points. First, the “hygiene hypothesis” states that the reduction of parasites and microorganisms hinders the maturation of the immune system [38]. In the process of coevolution between worms and their hosts, a delicate mechanism for regulating the host's immune system is formed [39]. The immune response induced by *T. spiralis* can inhibit tumour growth [24]. Second, *T. spiralis* can induce tumour cell apoptosis. Apoptosis plays an important role in antitumour immunity. A study showed that downregulation of apoptosis leads to unrestricted cell proliferation connected with the occurrence and development of tumours and drug resistance [40]. Thus, it is an essential approach for treating tumours to restore or enhance the sensitivity of cancer cells to apoptosis, and has been highlighted as a new strategy for modern tumour therapy [41]. Studies have shown that *T. spiralis* can cause apoptosis of B16 melanoma in animal models [36], and its protein can induce apoptosis of K562 and H7402 cells [21, 25]. Third, there are cross antigens between murine-derived tumours and *T. spiralis* that have significant antitumour effects. However, cross antigens between human-derived tumours and *T. spiralis* have not yet been reported.

In this study, cross antigen TPD52 between human osteosarcoma and *T. spiralis* was identified. The* TPD52 *gene, first found in breast cancer, is in the core region of chromosome 8q21 amplification [42]. Therefore, TPD52 is a novel, potentially important tumour-associated antigen that is highly expressed in a variety of tumour cells, affecting their biological function by regulating their proliferation, apoptosis, invasion, and migration [43–45].

This study found that anti-TPD52 antiserum has a curative effect on osteosarcoma in vivo. The tumour suppression rate may increase with prolonged treatment; however, the tumour diameter was too large, and the experiments had to be ended to comply with animal ethical guidelines. Current results showed that anti-TPD52 antiserum is more effective than anti-*T. spiralis* antiserum, possibly because the specificity of TPD52 is higher than that of *T. spiralis*, and the prepared antiserum has stronger targeting ability.

Our preliminary studies have shown that anti-TPD52 antiserum can exert anti-osteosarcoma effects by promoting the secretion of antitumour cytokines. Although nude mice lack T cells, some natural immune cells such as macrophages, natural killer cells, and dendritic cells can also secrete cytokines [46]. TNF-α and IFN-γ are two important pro-inflammatory cytokines with multiple biological functions. Both TNF-α and IFN-γ can cause tumour cell necrosis by destroying blood vessels and can also directly promote tumour cell apoptosis [47–49]. IL-10 also has antitumour activity and can induce cytotoxicity of CD8+ T cells. In mouse tumour models, IL-10 inhibits tumour growth by increasing the expression of IFN-γ in CD8+ T cells [50]. IL-12 can also kill primary and metastatic tumours by inducing T helper 1 (Th1) response and enhancing CD8+ T cell response [51, 52].

We also found that anti-TPD52 antiserum can exert an anti-osteosarcoma effect by inducing apoptosis. The biological activity of antibody therapy is not only mediated by the natural immune effector mechanisms but is also related to apoptosis. Some monoclonal antibodies for the treatment of haematological tumours including rituximab [53] and tositumomab [54] can directly induce tumour cell apoptosis.

Antibody therapy has achieved remarkable efficacy, and several monoclonal antibodies, such as panitumumab [55] and cetuximab [56], have shown clinical efficacy. But antibodies can also cause side effects, including mild symptoms such as gastrointestinal symptoms, mucositis, and myelosuppression [57], as well as life-threatening adverse reactions such as cytokine release syndrome [58], central nervous system toxicity [59], immune-related adverse events [60], and inhibition of angiogenesis [61]. The occurrence of these side effects is related not only to the dose used but also to the route of vaccination. Studies have shown that the incidence of adverse reactions after subcutaneous vaccination is much lower than that with intravenous injection [62], but these studies did not consider individual differences, and the sample size was insufficient. This study found that anti-TPD52 antiserum did not cause significant pathological damage in nude mice, laying the foundation for further clinical applications.

This study initially found that anti-TPD52 antiserum inhibited the growth of osteosarcoma by inducing apoptosis and enhancing immunity. Determining the detailed mechanisms in the process, such as the roles of the innate immune or complement systems, will require further studies.

## Conclusions

Antiserum against the cross antigen TPD52 between osteosarcoma and *T. spiralis* can inhibit osteosarcoma by inducing apoptosis, without causing distinct pathological damage.

## Supplementary Information

**Additional file 1: Table S1.** Histopathological score criteria for heart, liver, spleen, lung, and kidneys.

**Additional file 2: Table S2.** Information on seven antigenic genes obtained by screening.

**Additional file 3: Table S3.** Anti-TPD52 antiserum titre detection (OD_450_).

**Additional file 4: Table S4.** Tumour volume, weight, and tumour inhibition rate (M ± SD).

## Data Availability

Data supporting the conclusions of this article are included within the article.

## Abbreviations

*T**. spiralis*: *Trichinella spiralis*
ML: Muscle larvae
TPD52: Tumour protein D52
ELISA: Enzyme-linked immunosorbent assay
HE staining: Haematoxylin and eosin staining
BCA: BCA protein assay kit
CFA: Complete Freund's adjuvant
*E. coli*: *Escherichia coli*
ORF: Open reading frame
ESAs: Excretory-secretory antigens
IPTG: Isopropyl β-d-thiogalactoside
CCK-8: Cell Counting Kit-8
LDH: Lactate dehydrogenase
