# Supplementary material for: Anti-osteosarcoma effect of antiserum against cross antigen TPD52 between osteosarcoma and Trichinella spiralis
Source: Parasit Vectors. 2021 Sep 26;14:498. doi: 10.1186/s13071-021-05008-6 (PMC8474799; doi:10.1186/s13071-021-05008-6)
Supplement: Supplementary file 1 — Additional file 1: Table S1. Histopathological score criteria for heart, liver, spleen, lung, and kidneys. [file 13071_2021_5008_MOESM1_ESM.pdf]

Additional file 1: Table S1. Histopathological score criteria for heart, liver, spleen, lung, and kidneys.

Tissues and organs	Scoring criteria	score
Heart	Inflammatory cell infiltration: none. Myocyte lesion: none.	0
	Inflammatory cell infiltration: mild (focal, perivascular) infiltration. Myocyte lesion: vacuolization (myocyte becomes vacuolized).	1
	Inflammatory cell infiltration: moderate (multifocal) infiltration. Myocyte lesion: focal myocyte necrosis (irregular border; fragmented sarcoplasm, debris, myocyte dropout).	2
	Inflammatory cell infiltration: extensive (most dense and diffuse) infiltration. Myocyte lesion: extensive myocyte necrosis (interstitial haemorrhage and eosinophil infiltration).	3
Liver	Minimal or no evidence of injury.	0
	Mild injury consisting in cytoplasmic vacuolation and focal nuclear pyknosis.	1
	Moderate to severe injury with extensive nuclear pyknosis, cytoplasmic hypereosinophilia, and loss of intercellular borders.	2
	Severe necrosis with disintegration of hepatic cords, hemorrhage, and neutrophil infiltration.	3
Spleen	Normal, no lymphocyte infiltration.	0
	Local infiltration of lymphocytes.	1
	More than 50% lymphocyte infiltration.	2
	Structure destruction of spleen.	3
Lung	Overdistension, septal/interstitial edema, alveolar exsudation: none. Extent of inflammation: none.	0
	Overdistension, septal/interstitial edema, alveolar exsudation: slight. Extent of inflammation: sparely.	1
	Overdistension, septal/interstitial edema, alveolar exsudation: medium. Extent of inflammation: ≤50% of the sample.	2
	Overdistension, septal/interstitial edema, alveolar exsudation: severe. Extent of inflammation: >50% of the sample.	3
Kidneys	No damage.	0
	Expansion of renal tubules.	1
	Focal necrosis of renal tubules.	2
	Extensive necrosis of renal tubules.	3
