# Supplementary material for: Anti-osteosarcoma effect of antiserum against cross antigen TPD52 between osteosarcoma and Trichinella spiralis
Source: Parasit Vectors. 2021 Sep 26;14:498. doi: 10.1186/s13071-021-05008-6 (PMC8474799; doi:10.1186/s13071-021-05008-6)
Supplement: Supplementary file 2 — Additional file 2: Table S2. Information on seven antigenic genes obtained by screening. [file 13071_2021_5008_MOESM2_ESM.pdf]

Additional file 2: Table S2. Information on seven antigenic genes obtained by screening.

Sequence ID	Protein	Open reading frame (bp)	Number of amino acids	Isoelectric point	molecular weight (KDa)	Grand average of hydropathicity	Predictive score of protective antigen
XM_003375331.1	*Trichinella spiralis* tumor protein D52	459	152	6.17	16.89	-0.767	0.7106
XM_003380156.1	*Trichinella spiralis* translationally-controlled tumor protein-like protein	633	210	4.77	24.22	-0.109	0.4393
XM_003377810.1	*Trichinella spiralis* transmembrane protein 106B	642	213	7.86	24	0.156	0.3363
DQ350146.1	*Trichinella pseudospiralis* translationally-controlled tumor protein	534	177	4.63	20.42	-0.412	0.4735
XM_003372136.1	*Trichinella spiralis* aminotransferase, classes I and II superfamily	1734	577	8.41	65.29	-0.258	0.5636
XM_003379699.1	*Trichinella spiralis* GTP-binding protein Ryh1	624	207	7.19	23.42	-0.398	0.5324
XM_003377681.1	*Trichinella spiralis* histone-arginine	3003	1001	5.05	112.53	- 0.116	0.5092

	methyltransferase
	CARM1

DNASTAR software was used to determine the open reading frame (ORF) of genes, and the isoelectric point and molecular weight of proteins. ProtParam tools were used to analyse the hydrophilicity of proteins and VaxiJen 2.0 was used to predict antigenicity.
