# Supplementary material for: Anti-osteosarcoma effect of antiserum against cross antigen TPD52 between osteosarcoma and Trichinella spiralis
Source: Parasit Vectors. 2021 Sep 26;14:498. doi: 10.1186/s13071-021-05008-6 (PMC8474799; doi:10.1186/s13071-021-05008-6)
Supplement: Supplementary file 3 — Additional file 3: Table S3. Anti-TPD52 antiserum titre detection (OD450). [file 13071_2021_5008_MOESM3_ESM.pdf]

Additional file 3: Table S3. Anti-TPD52 antiserum titer detection (OD450).

Groups	Serum dilution						
	1: 100	1: 400	1:1600	1:6400	1: 25600	1: 102400	1: 204800
Negative serum	0.176	0.163	0.128	0.199	0.070	0.058	0.063
Immune serum 1	0.877	0.839	0.760	0.599	0.327	0.153	0.114
Immune serum 2	0.894	0.785	0.688	0.501	0.259	0.123	0.093
Immune serum 3	0.838	0.811	0.733	0.531	0.283	0.136	0.094
Immune serum 4	0.909	0.856	0.778	0.599	0.364	0.170	0.119
Immune serum 5	0.963	0.928	0.864	0.710	0.430	0.217	0.127
Immune serum 6	0.990	0.941	0.883	0.688	0.416	0.192	0.123
Immune serum 7	1.045	0.948	0.873	0.704	0.422	0.204	0.126
Immune serum 8	0.970	0.941	0.834	0.651	0.414	0.190	0.114
Immune serum 9	1.018	0.937	0.794	0.653	0.448	0.221	0.129
Immune serum 10	0.995	0.857	0.665	0.527	0.348	0.226	0.126

Positive standard: $OD_{Immune} / OD_{Negative} > 2.1$.
