# Supplementary material for: Anti-osteosarcoma effect of antiserum against cross antigen TPD52 between osteosarcoma and Trichinella spiralis
Source: Parasit Vectors. 2021 Sep 26;14:498. doi: 10.1186/s13071-021-05008-6 (PMC8474799; doi:10.1186/s13071-021-05008-6)
Supplement: Supplementary file 4 — Additional file 4: Table S4. Tumour volume, weight, and tumour inhibition rate (M ± SD). [file 13071_2021_5008_MOESM4_ESM.pdf]

Additional file 4: Table S4. Tumour volume, weight, and tumour inhibition rate (M ± SD).

Groups (each group, n=5)	Tumor volume (mm^3) inhibition rate (%)	Tumor weight (g) inhibition rate (%)
PBS	986.5±64.7	1476.4±118.3
Negative serum	869.9±54.8 (11.82)	1335.8±64.0 (9.52)
100 μg anti-*T. spiralis* antiserum	546.7±100.9**** (44.58)	854.2±69.9**** (42.14)
25 μg anti-TPD52 antiserum	662.4±82.4**** (32.85)	972.4±70.4**** (34.14)
50 μg anti-TPD52 antiserum	423.4±29.7**** (57.08)	673.6±39.0**** (54.38)
100 μg anti-TPD52 antiserum	375.4±32.5**** (61.95)	597.0±59.5**** (59.56)

PBS: osteosarcoma challenge model. NS: negative serum. TS: 100 μg anti-*T. spiralis* antiserum. 25, 50, and 100 μg: anti-TPD52 antiserum.

(****P<0.0001 vs PBS).
